# RNABindRPlus: A Predictor that Combines Machine Learning and Sequence Homology-Based Methods to Improve the Reliability of Predicted RNA-Binding Residues in Proteins

**DOI:** 10.1371/journal.pone.0097725

**Published:** 2014-05-20

**Authors:** Rasna R. Walia, Li C. Xue, Katherine Wilkins, Yasser El-Manzalawy, Drena Dobbs, Vasant Honavar

**Affiliations:** 1 Bioinformatics and Computational Biology Program, Iowa State University, Ames, Iowa, United States of America; 2 Department of Computer Science, Iowa State University, Ames, Iowa, United States of America; 3 College of Information Sciences and Technology, Pennsylvania State University, University Park, Pennsylvania, United States of America; 4 Department of Plant Pathology and Plant-Microbe Biology, Cornell University, Ithaca, New York, United States of America; 5 Graduate Field of Computational Biology, Cornell University, Ithaca, New York, United States of America; 6 Department of Systems and Computer Engineering, Al-Azhar University, Cairo, Egypt; 7 Department of Genetics, Development, and Cell Biology, Iowa State University, Ames, Iowa, United States of America; 8 Bioinformatics and Genomics Graduate Program, Pennsylvania State University, University Park, Pennsylvania, United States of America; 9 The Huck Institutes of the Life Sciences, Pennsylvania State University, University Park, Pennsylvania, United States of America; University of Alberta, Canada

## Abstract

Protein-RNA interactions are central to essential cellular processes such as protein synthesis and regulation of gene expression and play roles in human infectious and genetic diseases. Reliable identification of protein-RNA interfaces is critical for understanding the structural bases and functional implications of such interactions and for developing effective approaches to rational drug design. Sequence-based computational methods offer a viable, cost-effective way to identify putative RNA-binding residues in RNA-binding proteins. Here we report two novel approaches: (i) HomPRIP, a sequence homology-based method for predicting RNA-binding sites in proteins; (ii) RNABindRPlus, a new method that combines predictions from HomPRIP with those from an optimized Support Vector Machine (SVM) classifier trained on a benchmark dataset of 198 RNA-binding proteins. Although highly reliable, HomPRIP cannot make predictions for the unaligned parts of query proteins and its coverage is limited by the availability of close sequence homologs of the query protein with experimentally determined RNA-binding sites. RNABindRPlus overcomes these limitations. We compared the performance of HomPRIP and RNABindRPlus with that of several state-of-the-art predictors on two test sets, RB44 and RB111. On a subset of proteins for which homologs with experimentally determined interfaces could be reliably identified, HomPRIP outperformed all other methods achieving an MCC of 0.63 on RB44 and 0.83 on RB111. RNABindRPlus was able to predict RNA-binding residues of all proteins in both test sets, achieving an MCC of 0.55 and 0.37, respectively, and outperforming all other methods, including those that make use of structure-derived features of proteins. More importantly, RNABindRPlus outperforms all other methods for any choice of tradeoff between precision and recall. An important advantage of both HomPRIP and RNABindRPlus is that they rely on readily available sequence and sequence-derived features of RNA-binding proteins. A webserver implementation of both methods is freely available at http://einstein.cs.iastate.edu/RNABindRPlus/.

## Introduction

Protein-RNA interactions play key roles in many vital cellular processes including translation [1], [2], post-transcriptional regulation of gene expression [3], [4], RNA splicing [5], [6], and viral replication [7], [8]. Recent evidence points to the role of non-coding RNAs (ncRNAs) in a number of human diseases [9]–[12] such as Alzheimer's [13], [14] and various cancers [15]–[18]. Reliable identification of protein-RNA interfaces is critical for understanding the structural bases, the underlying mechanisms, and functional implications of protein-RNA interactions. Such understanding is essential for the success of efforts aimed at identifying novel therapies for genetic and infectious diseases.

Despite extensive structural genomics efforts, the number of solved protein-RNA structures substantially lags behind the number of possible protein-RNA complexes [19]. Because of the cost and effort involved in the experimental determination of protein-RNA complex structures [20], [21] and RNA-binding sites in proteins [22], [23], considerable effort has been directed at developing reliable computational methods for predicting RNA-binding residues in proteins.

Computational approaches to protein-RNA interface prediction fall into two broad categories [19], [24]: (i) Sequence-based methods, which use an encoding of sequence-derived features of a target residue and its neighboring residues in sequence (sequence neighbors) to make predictions, and (ii) Structure-based methods, which use an encoding of structure-derived features of a target residue and its neighboring residues in sequence or structure to make predictions. Sequence-based methods [25]–[36] have exploited features such as amino acid sequence identity, physicochemical properties of amino acids, predicted solvent accessibility, position-specific scoring matrices (PSSMs), and interface propensities, among others. Structure-based methods [37]–[41] have used features such as amino acid doublet propensities of surface residues, geometry (patches or clefts) of the protein surface, roughness, and atomic protrusion (CX) values, to make predictions of RNA-binding residues in proteins.

Two recent comprehensive surveys of machine learning methods for predicting interfacial residues in protein-RNA complexes [19], [24] came to a somewhat surprising conclusion that the performance of sequence-based methods, especially those that use PSSMs to encode protein sequences, is comparable to that of structure-based methods, i.e., methods that take advantage of three-dimensional structure of the target protein, when available. 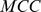 (Matthews Correlation Coefficient) values for the best methods ranged from 0.38 to 0.46. The difference in performance of the best performing methods was relatively small, and in several cases, not statistically significant [24].

Homology-based methods have proven successful in many bioinformatics tasks, including protein structure prediction [42], protein function annotation [43], [44], protein interaction prediction [45], protein-protein docking [46], [47] and protein-protein interface prediction, based on either sequence homology [48] or structure homology [49]–[52]. Homology-based methods have been shown to outperform other methods whenever close sequence or structural homologs of query proteins (used as templates) can be reliably identified [48], [49], [53]. Based on their analysis of a dataset of 261 protein-RNA complexes, Spriggs and Jones [54] concluded that RNA-binding residues are more conserved than other surface residues in RNA-binding proteins. To the best of our knowledge, however, there have been no studies that have examined the extent to which RNA-binding residues are indeed conserved among homologous proteins, or used sequence homology to reliably predict RNA-binding residues in protein.

Against this background, we explore whether sequence homology can be used to accurately predict RNA-binding residues in proteins and whether the resulting sequence homology-based approach can be combined with a state-of-the-art machine learning method to enhance the reliability of the predicted RNA-binding residues. Specifically, we: (i) introduce a novel sequence homology-based approach for prediction RNA-binding residues in proteins, HomPRIP, which accurately predicts the RNA-binding residues in a query protein based on the known RNA-binding residues of sequence homologs of the query protein (whenever such homologs are available); and (ii) propose RNABindRPlus, a novel two-stage predictor that uses logistic regression to optimally combine the predictions from HomPRIP and an optimized SVM classifier, SVMOpt, trained to predict RNA-binding interface residues using only sequence derived features of the query protein. We demonstrate that RNABindRPlus substantially outperforms existing sequence-based and structure-based methods. Both HomPRIP and RNABindRPlus have been implemented in a webserver that can be used to reliably predict RNA-binding residues in proteins, even when the structure of the query protein is unavailable.

## Results and Discussion

### Rationale for Homology-Based Approach

If RNA-binding residues are conserved across homologous proteins, we can use a simple sequence homology-based approach to predict RNA-binding residues in a query protein: Identify close sequence homologs of the query protein; infer the RNA-binding residues of the query protein based on the known RNA-binding residues of homolog(s) that are aligned with the query protein. The greater the extent to which RNA-binding residues are conserved across homologous protein-RNA complexes, the greater is the reliability with which the RNA-binding residues of a query protein can be predicted based on the known RNA-binding residues of its sequence homologs.

### Conservation Analysis of RNA-Binding Residues in Protein-RNA Complexes

Following the approach of Xue et al. [48], we define an interface conservation score 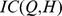 that measures the correlation between the interface (and non-interface) residues of a query protein 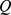 and its putative sequence homolog 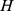 when the two are aligned (see Methods for details). The 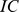 score measures the degree to which RNA-binding residues of 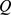 are conserved in (and hence can be predicted from the known interface residues of) the protein 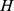. We calculated the pairwise 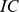 scores of proteins in a non-redundant dataset of 216 RNA-binding proteins (RBPs) extracted from the PDB (Protein Data Bank, [55]) as of October 2010 (NR216, see Methods). Our analysis showed that RNA-binding residues of a protein are highly conserved among its close sequence homologs (data not shown).

Whenever a query protein has a sufficiently high 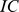 score with respect to its putative sequence homolog, we can predict its RNA-binding residues based on the known RNA-binding residues of its sequence homolog. However, examination of the precise definition of the 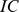 score of a protein with respect to its putative sequence homolog (see Methods) shows that computing it requires knowledge of the RNA-binding residues of both the query protein and its homolog. How can we then use the 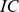 score, 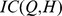, of a query protein 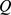 with respect to a putative sequence homolog 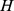 to determine whether we can reliably *predict* the *unknown* RNA-binding residues of 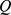 based on the *known* RNA-binding residues of 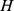
? Fortunately, as shown below, we can estimate 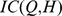 using available information, e.g., the sequence alignment of 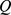 with 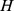. Specifically, we construct a regression model to predict the 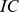 score for the query protein from its alignment with its sequence homolog(s) with known RNA-binding residues.

### Predicting the Interface Conservation Score of a Query Protein

**Figure 1:**
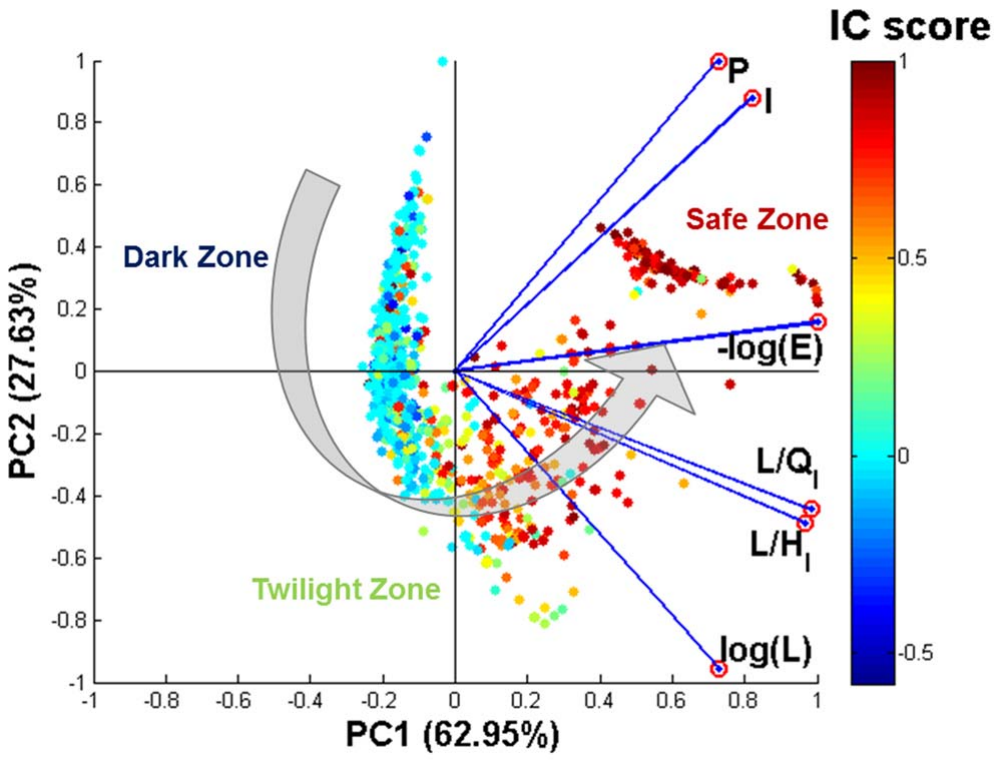
Principal Components Analysis (PCA) of interface conservation scores and sequence alignment statistics. Data points in the plot correspond to the projection of a 6-dimensional vector representing the pairwise alignment of a query and homolog sequence onto a 2-dimensional space defined by the first and second principal components. Blue lines with red circles at their tips represent the axes of the original 6-dimensional space for the 6 variables used in PCA analysis: -log(E) (where 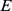 is the 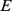-value), Identity Score (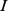), Positive Score (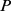), log(L) (where 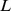 is local alignment length), alignment length fractions (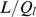 and 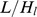, where 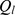 and 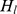 are the lengths of the query and homolog proteins, respectively). Each data point is colored according to its computed 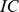 score, with higher 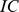 score (red/orange) indicating higher interface conservation and lower 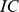 scores (blue/green) indicating lower interface conservation. The large gray arrow indicates the direction of increasing degree of interface conservation, from Dark to Twilight to Safe Zone.

We used Principal Components Analysis (PCA) to explore the relationship between six key sequence alignment statistics (see Methods), that are indicative of the quality of the alignment of a protein with its putative sequence homologs, and the IC score of the protein. Our analysis showed that a large fraction (90.6%) of the variance of the IC score is accounted for by the first two principal components. Figure 1 shows the projection of 6-dimensional alignment statistics of a protein and its sequence homolog(s) onto a 2-dimensional plane defined by the first two principal components. The resulting 2-dimensional interface conservation space can be partitioned into three regions based on the IC score: (i) Dark Zone, which contains query-homolog pairs with low IC scores (blue data points); (ii) Twilight Zone, which contains query-homolog pairs with intermediate IC scores (yellow, orange, and green data points); and (iii) Safe Zone, which contains query-homolog pairs with high IC scores (red data points).

Based on the results of the PCA analysis which shows that the *Positive Score* and *Identity Score* (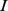) are highly correlated with each other, we chose to include only the Positive Score (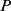) along with 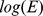, 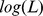 (where 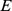 is the 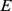-value, 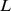 is the Local Alignment Length), and 
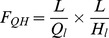
 (where 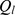 and 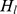 are lengths of the query protein 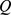 and its homolog 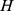, respectively) in the regression model that predicts the 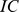 score 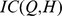: 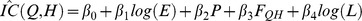



All the parameters (Table 1) of the regression model are significant (p-values 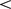 0.0001) and the model has an adjusted 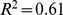. 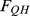 explains the largest fraction of Type II SS (Sum of Squares) error in the predicted 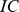 score and hence is a good proxy for the 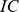 score.

**Table 1:** The Linear Model for Interface Conservation.

Variable	Parameter stimate	Standard Error	Type II SS
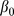	−0.532	0.042	8.70
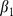	0.001	0.000	1.11
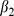	0.005	0.000	12.54
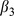	0.600	0.014	97.55
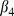	0.089	0.007	8.60

### HomPRIP: A Sequence Homology-Based RNA-Binding Site Predictor

Now that we have a means of predicting the 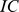 score, 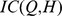, of a query protein 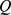 with respect to its putative sequence homolog 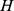 from the BLAST alignment scores of 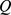 with 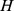, we can proceed to use the predicted 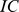 scores to choose homologs of the query protein to be used to infer the unknown RNA-binding residues of the query protein. HomPRIP, our sequence homology-based protein-RNA interface predictor operates as follows: Given a query protein 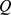, HomPRIP uses a BLAST search against the proteins in the Protein-RNA Interface Database [56], PRIDB, to identify a set of sequence homologs of 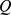, 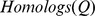, with known RNA-binding residues. Each sequence homolog 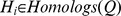 is assigned a weight 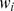, which is the predicted 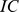 score, 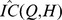. A weighted nearest neighbor classifier is used to infer the RNA-binding residues of the query protein based on the known interface residues of its closest homologs (see Methods). The reliability of the predicted RNA-binding residues in each case can be estimated based on the predicted 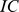 scores of the homologs used to arrive at the prediction.

### Evaluation of HomPRIP Predictions: Reliability and Coverage

In previous work, we used RB198, a non-redundant dataset of protein-RNA complexes [56] to assess the performance of alternative approaches to predicting RNA-binding residues in proteins [24]. For the purpose of comparison with previous approaches, we used each of the proteins in the RB198 dataset as a query protein to HomPRIP. HomPRIP searched for putative sequence homologs of the query proteins in RB198 against the nr_RNAprot_s2c database (see Datasets). Homologs that shared greater than 95% sequence similarity with the query proteins were discarded. This ensures that the query protein itself is excluded from being one of the homologs. HomPRIP was able to find at least one Safe, Twilight, or Dark Zone homolog for only 152 out of the 198 proteins in the RB198 dataset. The prediction performance of HomPRIP was evaluated using several standard metrics (see Methods for details). As shown in Table 2, for 45% of proteins in RB198, HomPRIP was able to find Safe Zone homologs and, as expected, very reliably predict their RNA-binding residues (with 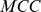 of 0.83, 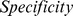 of 0.87, and 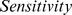 of 0.85). For 27% of the proteins, HomPRIP could find only Twilight Zone homologs and for 5%, only Dark Zone homologs. When predictions are based only on Twilight Zone homologs, the performance of HomPRIP drops to an 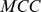 of 0.5, 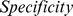 of 0.64, and 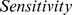 of 0.49. When predictions are based only on Dark Zone homologs, HomPRIP has an 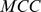 of 0.17, 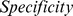 of 0.37, and 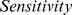 of 0.12. On the 152 proteins that had at least one homolog (from any zone), HomPRIP was able to predict RNA-binding residues with an 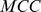 of 0.69, 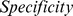 of 0.79, 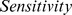 of 0.69, and an 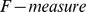 of 0.73.

**Table 2:** Performance of HomPRIP on RB198.

Homology Zone	Prediction Coverage	Specificity	Sensitivity	F-measure	MCC
Safe Zone	89/198 = 45%	0.87	0.85	0.86	0.83
Twilight Zone	54/198 = 27%	0.64	0.49	0.55	0.50
Dark Zone	9/198 = 5%	0.37	0.12	0.18	0.17
All Zones	152/198 = 77%	0.79	0.69	0.73	0.69

The performance is shown for the Safe, Twilight, and Dark Zones, separately. Prediction coverage is the fraction of queries that can be predicted by HomPRIP in a given zone.

**Figure 2:**
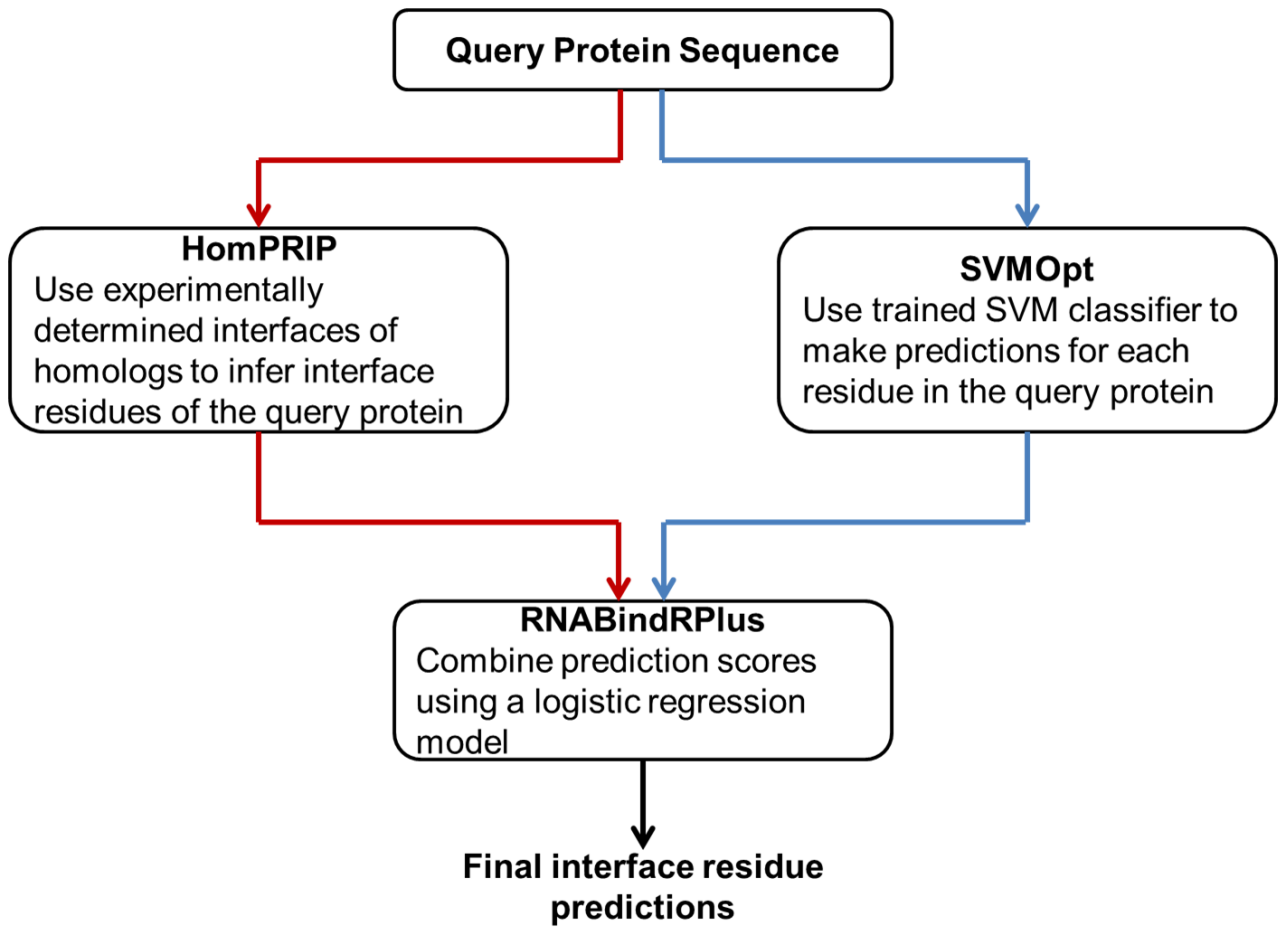
RNABindRPlus flowchart. Flowchart showing the different components of RNABindRPlus.

The prediction coverage of any sequence homology-based method for predicting RNA-binding residues of proteins is limited by the availability of homologs with known RNA-binding residues. Thus, HomPRIP fails to predict RNA-binding residues of query proteins that do not have at least one homolog with experimentally determined RNA-binding residues. For this reason, HomPRIP fails to return any predictions for 23% of proteins in the RB198 dataset. In addition, HomPRIP cannot make predictions on parts of a query protein sequence that are not aligned with any of its homologs. On the other hand, predictors trained using machine learning offer 100% coverage, although the increased coverage may come at the expense of the reduced reliability of predictions. To explore whether improved predictions can be obtained by combining a sequence homology-based method with a machine learning method, we developed RNABindRPlus, a hybrid predictor that combines HomPRIP predictions with those from an optimized Support Vector Machine (SVM) classifier, SVMOpt (Figure 2).

### Hybrid Method: RNABindRPlus

A recent study [24] compared the performance of Naïve Bayes (NB) and Support Vector Machine (SVM) classifiers trained to predict RNA-binding residues of proteins, from features of a sliding window of 25 amino acid residues centered on the target residue, using three different sequence-based feature representations (amino acid identity, position specific scoring matrices, and smoothed PSSMs [26]). The study concluded that an SVM classifier, SVM-RBF, which used a radial basis function (RBF) kernel and a PSSM profile to encode the target residue and its sequence neighbors, outperformed all other sequence-based RNA-binding site predictors and was competitive with predictors that use structure-derived features. The study used the default parameters (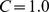 and 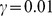) for the RBF kernel. In the current study, we used an optimized version of the SVM classifier, which we refer to as SVMOpt. The SVM classifier utilized by RNABindRPlus has the hyper parameters, 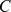 and 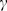, as well as the window size optimized (see Methods) for performance on the RB198 dataset. The best combination of parameters was found to be 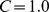, 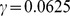 and a window size of 21 (data not shown). To predict whether or not a given amino acid is an RNA-binding residue, RNABindRPlus combines the prediction scores from HomPRIP with SVMOpt using a logistic regression classifier.

### Performance of HomPRIP and RNABindRPlus

To rigorously compare the performance of HomPRIP and RNABindRPlus with each other and with available state-of-the-art methods (see below), we used two independent test sets:

- RB44 [19] (see Datasets), an independent benchmark test set of 44 protein chains extracted from protein-RNA complexes deposited in the PDB between January 2011 and April 2011. The performance of a variety of methods for predicting RNA-binding residues in proteins was benchmarked on this dataset by Puton et al. [19]. Note that the datasets RB198 and RB44 share no common members.
- RB111, a more recently generated test set of 111 protein chains extracted from protein-RNA complexes deposited in the PDB between June 2010 to December 2010, and May 2011 to March 2014. Sequences in RB111 share less than 40% sequence similarity with sequences in RB198 and RB44.

Out of the 44 proteins in the RB44 dataset, HomPRIP was able to make predictions on 28 proteins. Table 3 compares the performance measures of different methods on these 28 proteins. HomPRIP achieved an 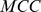 of 0.63 as compared to RNABindRPlus, which had an 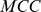 of 0.60 and the Metapredictor [19] and PiRaNhA [31], both of which had an 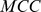 of 0.51. Other sequence- and structure-based methods tested had even lower values of 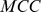. This result shows that when HomPRIP can identify homologs with known interfaces, it can outperform other methods.

**Table 3:** Evaluation of Methods on 28 proteins from the RB44 dataset.

Method	Reference	Specificity	Sensitivity	F-measure	MCC
HomPRIP	This paper	0.84	0.62	0.71	0.63
RNABindRPlus	This paper	0.76	0.67	0.71	0.60
SVMOpt	This paper	0.58	0.72	0.64	0.48
Metapredictor	[19]	0.74	0.54	0.62	0.51
PiRaNhA	[60]	0.66	0.65	0.65	0.51
BindN+	[35]	0.56	0.75	0.64	0.47
PPRInt	[29]	0.49	0.77	0.60	0.39
PRBR	[30]	0.58	0.45	0.51	0.34
RNABindR	[70]	0.60	0.39	0.48	0.32
BindN	[34]	0.50	0.50	0.50	0.28
NAPS	[25]	0.43	0.58	0.49	0.23
KYG**	[37]	0.55	0.66	0.60	0.41
OPRA**	[39]	0.61	0.48	0.53	0.37
PRIP**	[38]	0.47	0.71	0.56	0.33

The first 11 methods are sequence-based methods. The last 3 methods are structure-based methods (indicated by **). Methods in each category are sorted in descending order of MCC. The highest value in each column is shown in bold font.

Out of the 28 proteins, HomPRIP found Safe Zone homologs for 11 proteins, Twilight Zone homologs for 15 proteins, and Dark Zone homologs for 2 proteins. Table 4 lists the proteins from RB28 that have homologs in the different homology zones. Not surprisingly, HomPRIP achieved the best results with 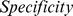, 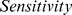, 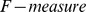, and 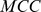 of 0.88, 0.80, 0.84 and 0.77, respectively on the 11 query proteins for which Safe Zone homologs could be found. On this subset of 11 proteins, HomPRIP substantially outperforms RNABindRPlus, which had 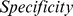, 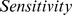, 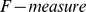, and 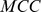 values of 0.79, 0.67, 0.72, and 0.61, respectively (Table 5). For 15 query proteins that had Twilight Zone homologs, HomPRIP had a higher 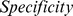 of 0.83 than RNABindRPlus (0.73). However, RNABindRPlus had higher values of 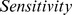, 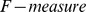, and 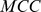 (Table 5). On the subset of 2 proteins that have Dark Zone homologs, RNABindRPlus achieved higher values of 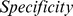, 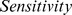, 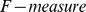, and 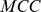 than HomPRIP (0.83, 0.54, 0.65, and 0.57 versus 0.45, 0.18, 0.26, and 0.13, respectively). Thus, although HomPRIP has higher values of performance metrics on query proteins that have Safe Zone homologs, RNABindRPlus has superior performance on query proteins that have homologs in the Twilight and Dark Zones.

**Table 4:** HomPRIP Performance by Zone on RB28.

Homology Zone	Proteins	Specificity	Sensitivity	F-measure	MCC
Safe Zone	2L5D_A, 2XD0_A, 2XZN_J, 3IZV_M, 3IZW_I, 3J00_G, 3J01_5, 3PIP_F, 3PIP_G, 3PIP_T, 3Q2T_A	0.88	0.80	0.84	0.77
Twilight Zone	2XXA_D, 2XZM_B, 2XZM_C, 2XZM_G, 2XZM_I, 2XZM_M, 3IZV_X, 2RRA_A, 2XZM_E, 2XZM_Q, 2XZN_L, 2XZM_8, 2XZM_S, 2XZM_U, 3IZW_R	0.83	0.55	0.66	0.58
Dark Zone	2XZM_D, 3PDM_P	0.45	0.18	0.26	0.13

All measures are highest for proteins with Safe Zone homologs and lowest for those with Dark Zone homologs.

**Table 5:** HomPRIP, RNABindRPlus, and SVMOpt Performance by Zone on RB28.

Safe Zone	Specificity	Sensitivity	F-measure	MCC
HomPRIP	0.88	0.80	0.84	0.77
RNABindRPlus	0.79	0.67	0.72	0.61
SVMOpt	0.63	0.68	0.65	0.48
**Twilight Zone**	**Specificity**	**Sensitivity**	**F-measure**	**MCC**
HomPRIP	0.83	0.55	0.66	0.58
RNABindRPlus	0.73	0.69	0.71	0.60
SVMOpt	0.54	0.76	0.63	0.47
**Dark Zone**	**Specificity**	**Sensitivity**	**F-measure**	**MCC**
HomPRIP	0.45	0.18	0.26	0.13
RNABindRPlus	0.83	0.54	0.65	0.57
SVMOpt	0.68	0.64	0.66	0.52

On the RB111 dataset, HomPRIP was able to make predictions on 49 proteins (Table 6). Table 7 compares the performance measures of different methods on these 49 proteins. Not surprisingly, HomPRIP achieves the highest values of all performance metrices on these 49 proteins (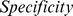 of 0.85, 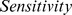 of 0.85, 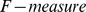 of 0.85 and 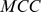 of 0.83), because it can find Safe Zone homologs for all of them. The second best method on this subset of RB111 is RNABindRPlus, achieving a 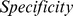 of 0.64, 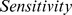 of 0.54, 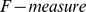 of 0.59, and 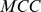 of 0.55.

**Table 6:** Proteins with Safe Zone Homologs in RB111.

Homology Zone	Proteins
Safe Zone	2XGJ_A, 2XS2_A, 2YSY_A, 3AGV_A, 3AMT_A, 3B0U_X, 3KFU_A, 3KFU_F, 3LWR_A, 3NMR_A, 3R2C_A, 3RC8_A, 3S14_A, 3S14_B, 3T5N_A, 3V22_V, 3V2C_Y, 3ZD6_A, 4AFY_A, 4ARC_A, 4ATO_A, 4B3G_A, 4BTD_2, 4BTD_D, 4BTD_G, 4BTD_S, 4BTD_X, 4DH9_Y, 4DWA_A, 4E78_A, 4ERD_A, 4IFD_A, 4IFD_H, 4K4Z_A, 4KJ5_5, 4KJ5_G, 3NVI_A, 3OIN_A, 3R9X_B, 3RW6_A, 3ULD_A, 3VYX_A, 4AM3_A, 4B3O_A, 4BA2_A, 4F02_A, 4F1N_A, 4FXD_A, 4GV3_A

There are 49 proteins in RB111 for which HomPRIP can find homologs and return predictions.

**Table 7:** Evaluation of Methods on 49 proteins from the RB111 dataset.

Method	Reference	Specificity	Sensitivity	F-measure	MCC
HomPRIP	This paper	**0.85**	**0.85**	**0.85**	**0.83**
RNABindRPlus	This paper	0.64	0.54	0.59	0.55
SVMOpt	This paper	0.27	0.51	0.35	0.28
BindN+	[35]	0.28	0.48	0.36	0.28
RNABindR v2.0	[24]	0.19	0.67	0.30	0.24
PPRInt	[29]	0.21	0.56	0.31	0.23
BindN	[34]	0.18	0.39	0.24	0.14
KYG**	[37]	0.20	0.46	0.28	0.19
PRIP**	[38]	0.19	0.49	0.27	0.19

The first 7 methods are sequence-based methods. The last 2 methods are structure-based methods (indicated by **). Methods in each category are sorted in descending order of MCC. The highest value in each column is shown in bold font.

These results confirm that HomPRIP's prediction performance is dependent upon the degree of sequence similarity between the query protein and its putative sequence homologs with known RNA-binding residues. More importantly, it demonstrates that the homology zones are good indicators of the reliability of HomPRIP's predictions. When Safe Zone homologs are available for query proteins, HomPRIP has the highest predictive performance. In contrast, the performance of RNABindRPlus is similar across proteins from different homology zones, although it is slightly lower than that of HomPRIP on query proteins in the Safe Zone.

### What Factors Lead to Superior Performance for RNABindRPlus?

As noted by Walia et al. [24], predictors that use PSSMs outperform those that use amino acid identity when evaluated using a standardized experimental setup (same datasets, same cross-validation procedure). Each score in a PSSM is a log-likelihood ratio of an amino acids appearance in a specific column of a multiple sequence alignment against a background distribution, representing the degree of conservation of the amino acid in that specific position; the higher the score, the higher the degree of conservation. Therefore, PSSMs capture important evolutionary information by exploiting the large number of available protein sequences, which are much easier to obtain than protein structures.

RNABindRPlus combines our homology-based method, HomPRIP, with SVMOpt, an optimized SVM classifier that uses a radial basis function (RBF) kernel with the sequence PSSM features. We believe that RNABindRPlus achieves a superior performance because it benefits from (i) the interface conservation information contributed by HomPRIP; (ii) residue conservation information encoded in PSSMs; and (iii) the hidden interaction patterns extracted by SVMOpt from the training set.

### Case Study: Accurate Identification of RNA-Binding Residues in the Human Immunorecognition Protein, RIG-I

RNA-protein interactions play key roles in the innate immune system in mammals, which is the first line of defense against invading viral and bacterial pathogens [57]. One class of cytosolic RNA-binding proteins, the RIG-I-Like receptors (RLRs), function as RNA sensors that can identify viral RNA as non-self by binding to specific molecular motifs in viral RNAs and activating cellular signaling pathways that stimulate host antiviral immune responses and suppress viral replication [58]. The crystal structure of the RIG-I C-terminal domain (CTD) bound to 5′pp dsRNA has been published [59], but was not included in the RB44 or RB198 benchmark datasets.

**Figure 3:**
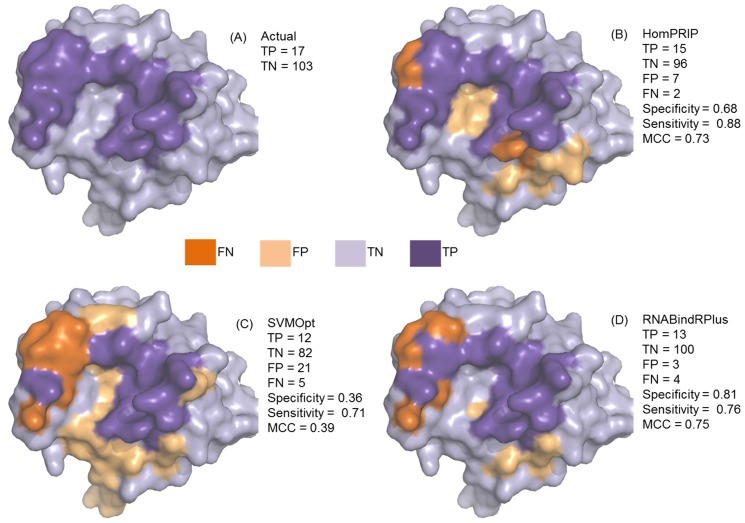
PDB ID: 3NCU, Chain A: RIG-I. (A) Actual interface residues, (B) Predictions made by HomPRIP, (C) Predictions made by SVMOpt, and (D) Predictions made by RNABindRPlus.

Figure 3 shows the predictions of HomPRIP, SVMOpt, and RNABindRPlus on the RIG-I CTD (PDB Id: 3NCU, chain A). All of the homologs used by HomPRIP for making the prediction were in the Safe Zone. This example illustrates how RNABindRPlus combines the predictions from HomPRIP and SVMOpt to provide better overall predictions. RNABindRPlus returns the lowest number of false positive predictions and has the highest 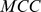 (0.75), compared to HomPRIP (0.73) and SVMOpt (0.39). RNABindRPlus also has the highest 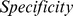 of 0.81 compared to HomPRIP (0.68) and SVMOpt (0.36) whereas HomPRIP has the highest 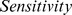 of 0.88 compared to RNABindRPlus (0.76) and SVMOpt (0.71). For many biological applications, high 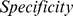 is desirable, because it allows researchers to identify a short list of residues for targeted mutations designed to alter the affinity or specificity of RNA-binding. As with most classifiers, RNABindRPlus can be tuned to favor even higher specificity, at the expense of lower sensitivity.

### RNABindRPlus Outperforms Other Predictors of RNA-binding Residues

On the RB44 dataset, we compared the performance of RNABindRPlus with eight sequence-based methods (see Table 8 for method descriptions) and three structure-based methods (see Table 9 for method descriptions). These methods were chosen based on a recent study [19] of the performance of readily available sequence- and structure-based predictors of RNA-binding sites in proteins. The Puton et al. study used webservers implementing these methods and concluded that the top performing sequence-based methods were a Metapredictor (which combines predictions from PiRaNhA, BindN+, and PPRInt), PiRaNhA [60], and BindN+ [35]. The top performing structure-based methods were KYG [37] and DRNA [41]. In our comparisons, we used the predictions returned by the same webservers (data shared with us by the Bujnicki group) with one exception. We did not compare our methods with the structure-based version of DRNA because the DRNA webserver uses structural homologs that may be exactly the same as the query protein, which could give the DRNA webserver an unfair advantage over other methods. DRNA can predict i) whether or not a protein is RNA-binding, and ii) which amino acids are RNA-binding. In the Puton et al. study, if a protein was predicted as non-RNA binding by DRNA, the case was considered to be one for which DRNA did not predict any RNA-binding residues [19]. However, in our experiments, we considered only the prediction of the RNA-binding residues, regardless of whether or not a protein was predicted to bind RNA. In addition, we included comparisons with another structure-based method, PRIP [38].

**Table 8:** Sequence-based Methods for Predicting RNA-binding sites in Proteins.

Method	Reference	Description
BindN	[34]	An SVM classifier that uses hydrophobicity, side chain pKa, molecular mass and PSSMs for predicting RNA-binding residues. It can also predict DNA-binding residues. Accessible at: http://bioinfo.ggc.org/bindn/
BindN+	[35]	An updated version of BindN, that uses an SVM classifier based on PSSMs and several other descriptors of evolutionary information. It can also predict DNA-binding residues. Accessible at: http://bioinfo.ggc.org/bindn+/
Metapredictor	[19]	A predictor that combines the output of PiRaNhA, PPRInt, and BindN+ to make predictions of RNA-binding residues using a weighted mean. Accessible at: http://iimcb.genesilico.pl/meta2/. The Metapredictor is not available as of March 2014.
NAPS	[25]	A modified C4.5 decision tree algorithm that uses amino acid identity, residue charge, and PSSMs to predict residues involved in DNA- or RNA-binding. Accessible at: http://prediction.bioengr.uic.edu/. The webserver cannot be accessed as of March 2014.
PiRaNhA	[60]	An SVM classifier that makes use of PSSM profiles, interface propensity, predicted solvent accessibility, and hydrophobicity to predict protein-RNA interface residues. Accessible at: http://bioinformatics.sussex.ac.uk/PIRANHA/. The webserver cannot be accessed as of March 2014.
PPRInt	[29]	An SVM classifier trained on PSSM profiles. Accessible at: http://www.imtech.res.in/raghava/pprint/
PRBR	[30]	An enriched random forest classifier trained on predicted secondary structure, a combination of PSSMs with physico-chemical properties, a polarity-charge correlation, and a hydrophobicity correlation. Accessible at: http://www.cbi.seu.edu.cn/PRBR/
RNABindR	[70]	A Naïve Bayes classifier that uses the amino acid sequence identity to predict RNA-binding residues in proteins. Previously accessible at: http://bindr.gdcb.iastate.edu/RNABindR/. It is no longer maintained.
RNABindR v2.0	[24]	An SVM classifier that uses sequence PSSMs to predict RNA-binding residues in proteins. Accessible at: http://einstein.cs.iastate.edu/RNABindR/.

**Table 9:** Structure-based Methods for Predicting RNA-binding sites in Proteins.

Method	Reference	Description
KYG	[37]	Uses a set of scores based on the RNA-binding propensity of individual and pairs of surface residues of the protein, used alone or in combination with position-specific multiple sequence profiles. Accessible at: http://cib.cf.ocha.ac.jp/KYG/. A stand-alone version of the method is also available.
OPRA	[39]	Uses patch energy scores calculated using interface propensity scores weighted by the accessible surface area of a residue to predict RNA-binding sites. The program is available upon request from the authors.
PRIP	[38]	Uses an SVM classifier and a combination of PSSM profiles, solvent accessible surface area (ASA), betweenness centrality, and retention coefficient as input features. Not accessible via the web server, but results can be obtained via correspondence with the author.

On the RB111 dataset, we compared the performance of RNABindRPlus with four sequence-based methods (BindN [34], BindN+ [35], PPRInt [29], and RNABindR v2.0 [24]) and two structure-based methods (KYG [37] and PRIP [38]). The Metapredictor [19], PiRaNhA [60], and NAPS [25] servers were all inaccessible at the time of running the experiments on RB111.

Because several methods return only binary predictions, we do not report Area under the ROC Curve (AUC) values, but instead compare the different methods based on 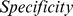, 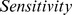, 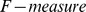 and 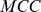.

**Figure 4:**
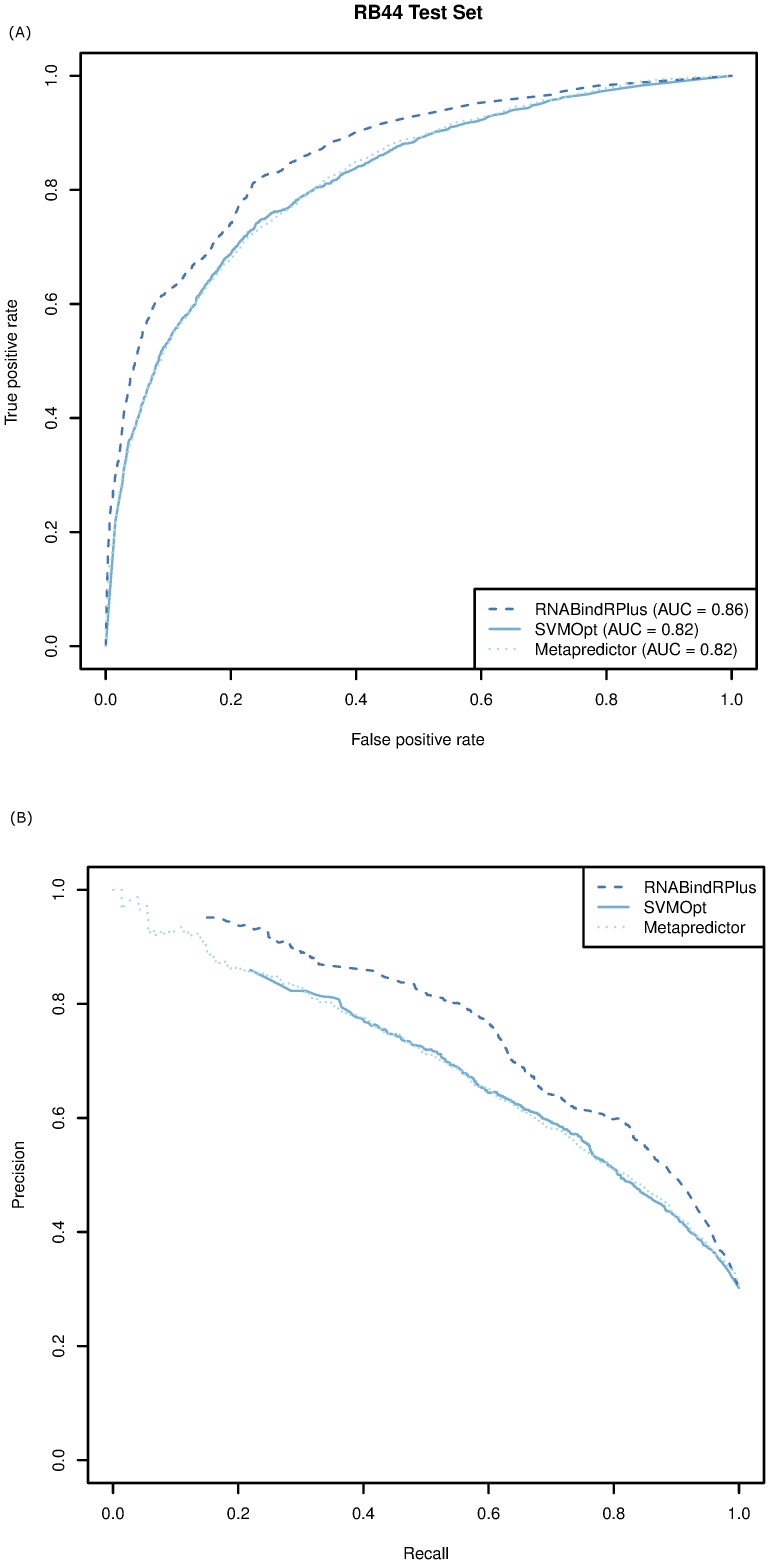
Comparison of SVMOpt, RNABindRPlus, and the Metapredictor on the RB44 dataset using (A) ROC curves and (B) PR curves with a 5 Å distance cut-off for interface residues.

The performance of different methods on the RB44 dataset is summarized in Table 10. Among all methods that return predictions for every query protein in the dataset (i.e., excluding HomPRIP), RNABindRPlus achieved the highest 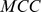 value of 0.55. The next highest 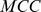 of 0.48 was obtained by PiRaNhA [60], and then by SVMOpt and the Metapredictor [19], both with an 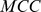 of 0.47. Notably, in terms of 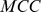, the best performing structure-based method was KYG [37] with a value of 0.42, considerably lower than the top sequence-based methods. The highest 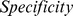 was obtained by the Metapredictor (0.74) followed by RNABindRPlus (0.72). The highest 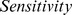 was obtained by BindN+ (0.73) [35] followed by SVMOpt and PPRInt [29] (0.72). RNABindRPlus had the highest 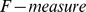 value of 0.67. A comparison of the ROC curves (Fig. 4a) shows that the performance of RNABindRPlus (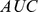 = 0.86) is superior to that of SVMOpt and the Metapredictor (both have an 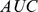 = 0.82). Similarly, the Precision-Recall (PR) curves (Fig. 4b) show that RNABindRPlus achieves a higher precision at all levels of recall than the other two methods.

**Table 10:** Evaluation of Methods on the RB44 dataset.

Method	Reference	Specificity	Sensitivity	F-measure	MCC
RNABindRPlus	This paper	0.72	0.63	**0.67**	**0.55**
SVMOpt	This paper	0.58	0.72	0.64	0.47
PiRaNhA	[60]	0.64	0.63	0.64	0.48
Metapredictor	[19]	**0.74**	0.49	0.59	0.47
BindN+	[35]	0.54	**0.73**	0.62	0.43
PPRInt	[29]	0.50	0.72	0.59	0.38
RNABindR	[70]	0.62	0.39	0.48	0.33
PRBR	[30]	0.58	0.41	0.48	0.31
BindN	[34]	0.50	0.51	0.50	0.28
NAPS	[25]	0.43	0.58	0.49	0.22
KYG**	[37]	0.56	0.67	0.61	0.42
OPRA**	[39]	0.57	0.51	0.54	0.36
PRIP**	[38]	0.46	0.68	0.55	0.31

The first 10 methods are sequence-based methods. The last 3 methods (indicated by **) are structure-based methods. Methods in each category are sorted in descending order of MCC. The highest value in each column is shown in bold font.

**Figure 5:**
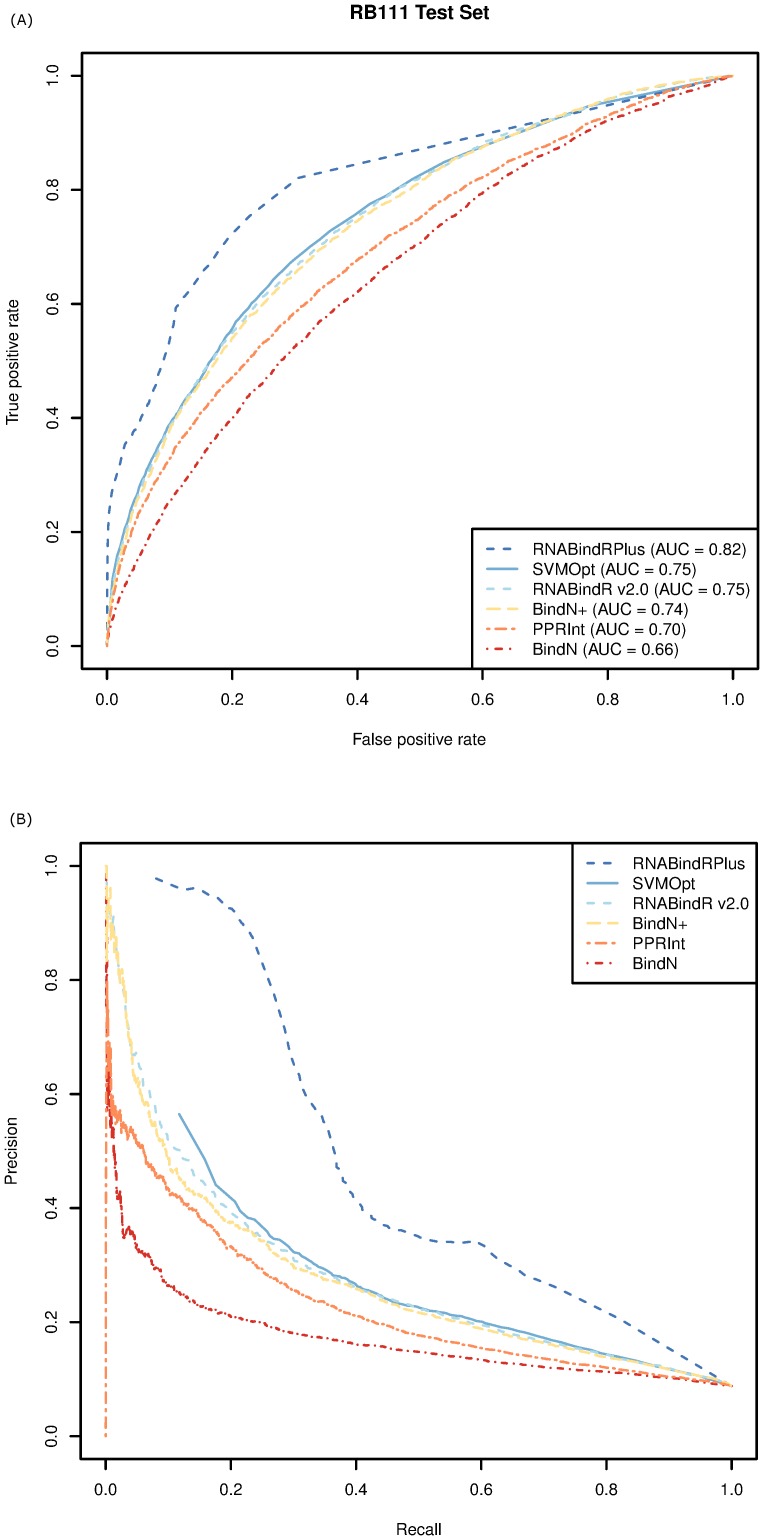
Comparison of SVMOpt, RNABindRPlus, RNABindR v2.0, BindN, BindN+ and PPRInt on the RB111 dataset using (A) ROC curves and (B) PR curves with a 5 Å distance cut-off for interface residues.

The performance of different methods on the RB111 dataset is summarized in Table 11. RNABindRPlus achieved the highest 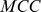 value of 0.37, followed by SVMOpt and BindN+ [35], both with an 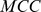 of 0.24. The best performing structure-based method on this dataset was KYG [37], with an 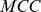 of 0.19, which is considerably lower than the top sequence-based methods. The highest 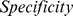 was obtained by RNABindRPlus (0.47) followed by a tie between SVMOpt and BindN+ [35] (0.25). The highest 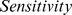 was obtained by RNABindR v2.0 [24] (0.63) followed by PPRInt [29] (0.48). RNABindRPlus had the highest 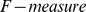 value of 0.37. A comparison of the ROC curves (Fig. 5a) shows that the performance of RNABindRPlus (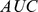 = 0.82) is superior to that of the other methods. Similarly, the PR curves (Fig. 5b) show that RNABindRPlus achieves a higher precision at all levels of recall than the other five methods.

**Table 11:** Evaluation of Methods on the RB111 dataset.

Method	Reference	Specificity	Sensitivity	F-measure	MCC
RNABindRPlus	This paper	**0.47**	0.37	**0.42**	**0.37**
SVMOpt	This paper	0.25	0.44	0.32	0.24
BindN+	[35]	0.25	0.43	0.31	0.24
RNABindR v2.0	[24]	0.18	**0.63**	0.28	0.22
PPRInt	[29]	0.18	0.48	0.26	0.18
BindN	[34]	0.16	0.39	0.23	0.14
KYG**	[37]	0.19	0.47	0.27	0.19
PRIP**	[38]	0.17	0.45	0.24	0.15

The first 6 methods are sequence-based methods. The last 2 methods (indicated by **) are structure-based methods. Methods in each category are sorted in descending order of MCC. The highest value in each column is shown in bold font.

Interestingly, the performance of all methods is better on the RB44 dataset than on the RB111 dataset. One possible explanation for this is that RB44 is composed mostly of ribosomal protein chains (36/44), whose roles are structural rather than enzymatic. In contrast, RB111 contains a much smaller proportion of ribosomal protein chains (10/111) and many more enzymes, including CRISPR nucleases, RNA helicases, and RNA methylases. This suggests that training custom classifiers on specific functional or structural classes of RNA-binding proteins could provide improved performance.

Taken together, these results demonstrate that the hybrid sequence-based method, RNABindRPlus, has substantially higher 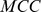 values than other methods evaluated here. Moreover, RNABindRPlus outperforms all other methods at any level of precision and recall. An unexpected result is that the top sequence-based methods, e.g., RNABindRPlus, BindN+, and SVMOpt, all have much higher 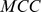 values than any of the structure-based methods.

### HomPRIP and RNABindRPlus Webservers

A webserver implementation of HomPRIP and RNABindRPlus is freely available at http://einstein.cs.iastate.edu/RNABindRPlus/. Users can submit a single or multiple proteins in FASTA format or upload a file containing proteins in FASTA format. Results returned include the RNA-binding residue predictions from HomPRIP, SVMOpt, and RNABindRPlus, as well as the prediction scores from each method. The server also returns a file containing the putative homologs and corresponding predicted 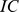 scores for the query protein(s). Users can utilize the 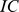 scores to determine whether their query protein(s) have Safe, Twilight, or Dark Zone homologs. A text file containing all potential homologs (i.e., the corresponding protein-RNA complexes with solved structures) and their sequence similarity to the query protein is also returned to the user.

## Materials and Methods

### Datasets

We utilized five datasets in our experiments.

1. nr_RNAprot_s2c: We built a BLAST database using RNA-binding proteins from PRIDB [56] (as of May 2013) with a resolution of 3.5 Å or better. There are 210,796 residues and 907 proteins in this database. In our experiments, this dataset was used with BLASTP-2.2.27+ [61] to search for putative sequence homologs.
2. NR216: We constructed a maximal non-redundant dataset of RNA-binding proteins (RBPs) using the following steps. We retrieved 9,649 protein chains from the set of all protein-RNA complexes in the PDB [55], [62] as of October 2010. Out of this redundant set of protein chains, we obtained 242 non-redundant protein chains using PISCES [63] with the following criteria: (i) sequence identity 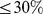; (ii) resolution of 3.5 Å or better; (iii) sequence length 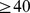 amino acids; (iv) non-X-ray entries were excluded; (v) CA-only entries were excluded. Further, we removed chains with interfaces containing fewer than 5 residues. An amino acid residue is considered an interface residue if it contains at least one heavy atom within 5 Å of any atom in the bound RNA. This definition of interface residues is used throughout this paper. The final dataset contained 216 non-redundant RBP chains with 8,420 interface residues and 48,129 non-interface residues (those residues that do not appear in the 3D structure of a complex are not counted, since we cannot determine if they are interface or not). We used NR216 for analyzing interface conservation in RNA-binding proteins.
3. RB198: RB199 [56] is a dataset that contains 199 non-redundant RNA-binding protein chains. It was created by using the PISCES server [63] to generate a set of proteins with 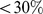 sequence identity and a resolution of 3.5 Å or better from all protein-RNA complexes in the PDB as of May 2010. To be included in the dataset, proteins must include 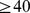 amino acids and 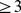 RNA-binding amino acids and the RNA in the complex must be 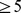 nucleotides long. RB198 is identical to RB199 except that one chain (2RFK_C) was omitted because it does not contain any interface residues based on the definition provided above. To maintain consistency with previous studies, both RB198 and RB199 include another chain (3EX7_A) which has no interface residues and one chain with only 2 interface residues (2J01_4). In this dataset, we consider residues that are not solved in the structure as non-interface residues. We used this dataset for cross-validation experiments and for training the final machine learning classifiers.
4. RB44: This is a non-redundant benchmark dataset compiled by Puton et al. [19] containing RNA-protein complexes deposited in the PDB [55], [62] between January 1st and April 28th 2011. It is composed of 44 protein chains that share 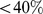 sequence identity. We used this dataset as an independent test set. None of the protein chains in RB44 share any global similarity with RB198 at a sequence similarity threshold of 40%.
5. RB111: This is a dataset compiled as of March 2014 that contains 111 non-redundant RNA-binding protein chains. It was created using the PISCES server [63] to generate a set of proteins with 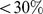 sequence identity and a resolution of 3.5 Å or better from all protein-RNA complexes deposited in the PDB between June 2010 and December 2010, and between May 2011 to March 2014. The dataset excludes any non-X-ray entries as well as CA-only entries. All protein chains in this dataset include 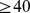 amino acids and 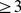 RNA-binding amino acids. We used this dataset as a newer, independent test set. None of the protein chains in RB111 share any global similarity with RB198 or RB44 at a sequence similarity threshold of 40% (tool used for this: CD-HIT [64], [65]).

### Sequence Conservation Analysis

We analyzed interface residues in structural homologs of each protein in a non-redundant dataset of 216 RNA-binding proteins, NR216. We extracted homologs for each of the 216 proteins from the nr_RNAprot_s2c database using BLASTP with an 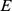 *-value* 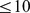. The structures and interface residues for proteins in NR216 and their homologs from nr_RNAprot_s2c were experimentally determined. From the resulting set of homologs, sequences that are likely to be copies of the query sequence and hence likely to introduce an undesirable bias in the estimation of sequence conservation were eliminated to obtain a dataset of 8,970 query/homolog pairs. For each query-homolog pair, (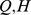), we calculated the interface conservation score, 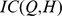, which is a measure of the degree of conservation of interface residues between the query protein, 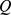 and its homolog(s), 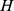. The higher the 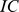 score, the more conserved are the interface residues between homologs and the query protein.

We studied the functional relationship of the 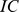 score with six alignment statistics, four of which are returned by BLAST [64] and two of which are derived from BLAST statistics: (i) *Positive score* (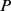), (ii) *Identity score* (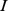), (iii) 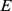 *-value* (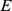), (iv) *Local Alignment Length* (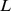), (v) 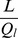 and (vi) 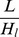 (where 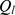 and 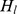 are lengths of the query protein 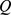 and its homolog 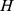, respectively). The last two measures tell us the extent of sequence homology between a query sequence, 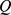 and its homolog, 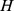. The 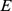-value is the expected number of random hits when a query sequence is searched against a database of a particular size. The smaller the E-value, the greater the chance that a hit is a biologically relevant homolog. *Identity score* measures the sequence identity shared by two amino acid sequences. BLASTP also returns a *Positive score* for a specific position, which calculates the observed substitutions that preserve the physicochemical properties of the original residue. A substitution of one residue type for another is labeled positive when the corresponding entry in the scoring matrix has a positive score. We represented each query-homolog alignment pair as a data point in a six-dimensional space defined by the six alignment statistics.

We used Principal Components Analysis (PCA), a dimensionality reduction technique, to visualize the relationship between the six sequence alignment statistics and the 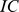 score. We also constructed a regression model to quantitatively describe interface conservation as a function of sequence alignment statistics.

### HomPRIP: A Sequence Homology-Based RNA-Binding Site Predictor

Given a query protein sequence, 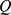, HomPRIP searches the nr_RNAprot_s2c database to identify homologous sequences that correspond to the protein components of experimentally determined protein-RNA complexes. The query protein itself is not utilized as one of the homologs. If at least one Safe Zone homolog is found, HomPRIP uses it to predict the interface residues of the query protein, 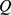. Otherwise, the search is repeated for homologs in the Twilight and Dark zones. HomPRIP reports the homology zones (Safe, Twilight, or Dark, see Table 12) accordingly, and uses the zone as an indicator of prediction confidence. Homologs that share 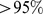 sequence identity with the query protein are discarded. This ensures conservative performance estimates for the method. If HomPRIP cannot find homologs in any of the three zones, it does not return any predictions for the query protein.

**Table 12:** Boundaries of Safe, Twilight, and Dark Zones used by HomPRIP.

Homology Zones	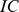 score Cutoff
Safe Zone	0.70
Twilight Zone	0.20
Dark Zone	0.15

HomPRIP assigns a prediction score to each residue of the query protein sequence based on the label of the residue in the corresponding position in its homolog(s) (after pairwise sequence alignment). Specifically, the prediction score (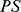) for the 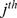 residue of the query protein is calculated as: 
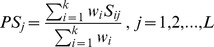
where 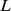 is the length of the query protein, 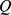 and 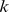 is the number of close homologs. 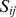 is the vote of a homolog 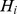 (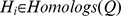) for the 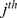 position of the alignment and is equal to 1 if the corresponding residue in the homolog is an interface residue and 0 otherwise. 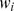 is 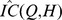, the 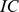 score predicted by the regression model for the 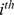 homolog of 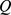. The prediction score, 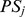, is converted into a binary prediction (1 represents an interface residue and 0 represents a non-interface residue) as follows:



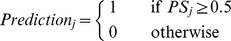



### SVMOpt: Support Vector Machine Classifier

From the Walia et al. [24] study, we picked the best performing feature, PSSMs, and the best classifier, SVM-RBF (SVM with the RBF kernel), and optimized the cost parameter C and the RBF kernel parameter, 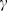, as well as the window sizes. We tuned these parameters using a three-dimensional grid search over the range 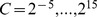 and 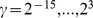 and window sizes ranging from 15 to 27. For finding the optimal values for 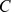, 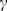, and the window size, we divided RB198 into training, validation, and test sets by splitting it into 6 parts. 165 chains were used for training and validation sets, and 33 chains were used as the held-out test set. Specifically, the optimization process was as follows: (i) Pick values for C, gamma, and the window size, (ii) Train the model using the training set, (iii) Evaluate the performance of the model on the validation set, (iv) Repeat steps (i)–(iii) using different training parameters, (v) Select the best model (parameter values) and train it using all the data from the training and validation sets, and (vi) Assess the final model using the held-out test set. Sequence-based 5-fold cross-validation was used in the optimization experiments, so steps (ii) and (iii) were repeated for each fold. We call the optimized classifier SVMOpt. The PSSMs were constructed by running PSI-BLAST [61] against the NCBI nr database for three iterations with an 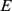-value cutoff of 0.001 for inclusion in the next iteration.

### Hybrid Method: RNABindRPlus

The prediction scores from HomPRIP and SVMOpt were combined using a second stage logistic regression model. The Weka implementation of logistic regression [66] was used with the default ridge parameter of 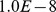. The input to the logistic regression model is a 2D vector representing the prediction scores from HomPRIP and SVMOpt. In cases where HomPRIP failed to return predictions (i.e., no homologs for query proteins are found or the target residue is not aligned with any residues in the homolog(s)), a missing input value (represented as ‘?’) is fed to the logistic regression model. We refer to this hybrid model as RNABindRPlus.

### Performance Evaluation

We used several different measures of classifier performance. On the RB198 dataset, performance measures were obtained by carrying out sequence-based 5-fold cross-validation. Sequence-based 5-fold cross-validation randomly divides protein chains in RB198 into 5 sets and alternatively uses 4 sets as the training set and 1 set as the test set. The average performance on the 5 test sets is used as the final evaluation of the classifier. Sequence-based cross-validation has been shown to be more rigorous than window-based cross-validation [67], because it ensures disjoint training and test sets at the sequence level instead of at the residue level. The predicted label for each residue is compared to the actual label and the residue is classified as a true positive (TP), false positive (FP), true negative (TN), or false negative (FN). We report the performance measures as defined in Baldi et al. [68].

Overall performance measures are calculated as follows: 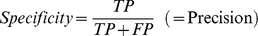


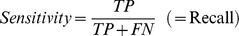


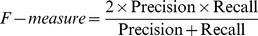


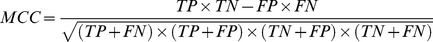



The measures describe different aspects of classifier performance. 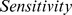 is the probability of correctly predicting the interface residues of a given protein. 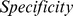 is the probability that a predicted interface residue in any given protein is in fact an interface residue. 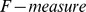 is the harmonic mean of precision and recall, where the best score is 1 and the worst score is 0. The *Matthews correlation coefficient* (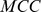) measures how predictions correlate with true interface and non-interfaces. All machine learning methods have an inherent trade-off between specificity and sensitivity that is controlled through the classification threshold. Predictors that make no positive predictions trivially achieve a 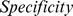 of 1. However, such methods are not useful, because they do not return any true positive predictions.

A *Receiver Operating Characteristic* (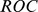) curve is useful for comparing classifiers across all classification thresholds. Where possible, we show the 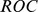 curve and report *Area under the ROC curve* (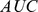). The 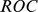 curve plots the proportion of correctly classified positive examples, *True Positive Rate* (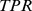), as a function of the proportion of incorrectly classified negative examples, *False Positive Rate* (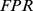), for different classification thresholds. When comparing the performance of two classifiers, for the same 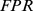, the one with a higher 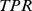 performs better. The ROCR package [69] in R was used to generate all 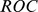 curves and *Precision-Recall* (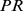) curves. When data are unbalanced (fewer interface residues than non-interface residues) 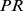 curves give a more informative picture of an algorithm's performance than 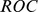 curves. In 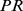 curves, we plot precision as a function of recall, with respect to different prediction score cutoffs. We also report the 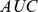 value, which is the probability that a classifier gives a higher score to a positive instance than to a negative instance. An 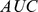 of 0.5 indicates a random discrimination between the positive and negative class while an 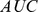 of 1.0 indicates perfect discrimination.

## Conclusions

We have shown that HomPRIP, a sequence homology-based method, can reliably predict RNA-binding residues when close sequence homologs of the query protein, with known RNA-binding residues, can be found. A sequence-based machine learning classifier, SVMOpt, returns reliable predictions for any query protein, regardless of whether structures of protein-RNA complexes containing homologous protein sequences are available. When Safe Zone homologs for a query protein can be found, HomPRIP is the method of choice. For other query proteins, RNABindRPlus, which combines HomPRIP with SVMOpt, has superior performance because it exploits the strengths of both methods. RNABindRPlus outperforms several state-of-the-art methods, both sequence-based and structure-based, for predicting RNA-binding sites in proteins. An important advantage of RNABindRPlus is that it is a purely sequence-based approach. A webserver implementation is freely available at http://einstein.cs.iastate.edu/RNABindRPlus/.
